# Concurrent Occurrence of Trichilemmal Cyst and Nevus of Ota: Case Series

**DOI:** 10.7759/cureus.52439

**Published:** 2024-01-17

**Authors:** Ramachandra Reddy Gowda Venkatesha, Karthik Rajaram Mohan, Vasu Sridhar Rao, Saramma Mathew Fenn, Reethika Rathan

**Affiliations:** 1 Oral Medicine and Radiology, Vinayaka Mission's Sankarachariyar Dental College, Vinayaka Mission's Research Foundation (Deemed to be University), Salem, IND

**Keywords:** q‐switched nd:yag laser, skin pigmentation, trigeminal nerve, trichilemmal cyst, nevus of ota

## Abstract

Nevus of Ota or congenital oculodermal melanosis (ODM) is characterized by brown or blue/gray asymptomatic brown or blue/gray flat lesions of the skin, mucosae, episcleral/sclera, and uvea, which are located near the trigeminal nerve's ophthalmic and mandibular branches. The main ophthalmic complications are glaucoma and predisposition to uveal melanoma. "trichilemmal cyst" is also known as "wen" "pilar cyst" or " isthmus catagen cyst". It occurs in the scalp and mimics sebaceous cysts clinically. The swelling appears smooth in outline and is filled with cytokeratin. An unusual case of a 32-year-old male with both trichilemmal cyst and nevus of Ota, a 27-year-old female, and a 47-year-old male with nevus of Ota is discussed here.

## Introduction

Nevus of Ota is a benign congenital, non-hereditary macular melanosis that primarily involves the region of the trigeminal nerve distribution [1]. The first division, namely the ophthalmic of the trigeminal nerve, and the second division, namely the maxillary, are most commonly affected [1]. Pilar cysts are appendageal cysts affecting the skin and are more common on the scalp [2]. They more often resemble sebaceous cysts [2]. The pilar cyst is a benign growth in the hair follicle, also known as the trichilemmal cyst [2]. Most cases occur on the scalp, though they can also occur in other areas of the head and neck region [2]. The incidence of pilar cysts is about 10% in the general population [2]. Most cases are sporadic and non-syndromic [2]. The pilar cysts are the most common types of skin cysts [2]. There is a higher incidence of these cysts in areas with a high concentration of hair follicles [2]. It is the most common cutaneous cyst on the scalp and the second most common on the head and neck [2]. In contrast to malignant lesions, pilar cysts are benign [2]. Approximately 2% of pilar cysts progress into malignant tumors, referred to as proliferating trichilemmal cysts or tumors [2]. It is possible to inherit trichilemmal cysts through autosomal dominant inheritance [2]. Typically, patients with familial pilar cysts are younger and have multiple lesions simultaneously [2]. These cysts develop from the epithelium between the sebaceous gland and the arrector pili muscle [2]. Most commonly, they occur on the head, especially the scalp [2]. It takes several years for the pilar cyst to grow to its full size; it grows very slowly [2].

## Case presentation

Case one

**Figure 1:**
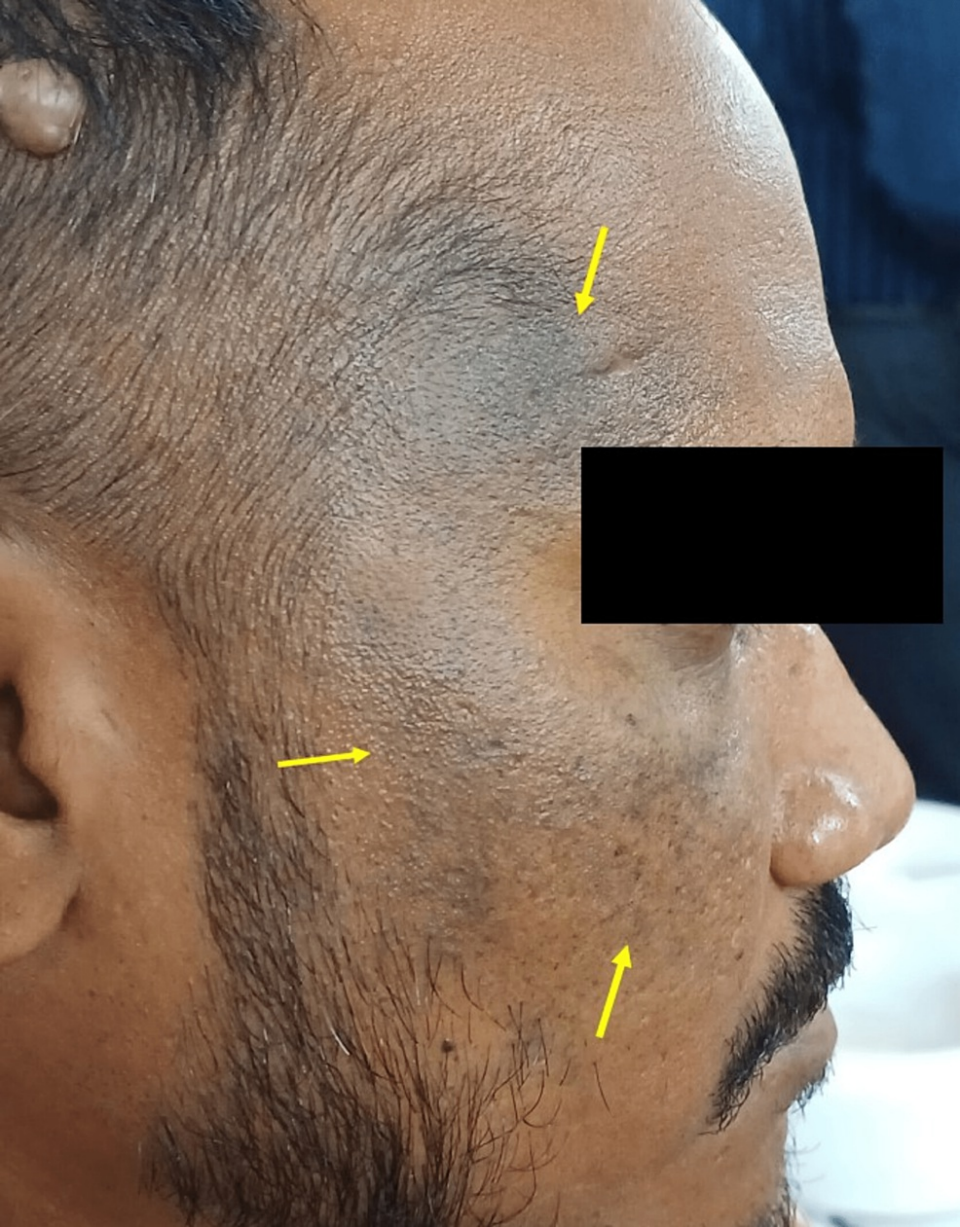
Extraoral examination revealed unilateral greyish pigmentations only involving the right forehead ( V1) and right cheek zygomatic (V2) regions on the face

A 32-year-old male reported to our dental outpatient department for a routine oral checkup. Extraoral examination revealed discrete macular greyish pigmentations present unilaterally only on the right forehead and cheek regions (Figure 1).

History revealed that these pigmentations are present from birth. The patient had no harmful habits of smoking or chewing tobacco. The patient had no medical history of diabetes, hypertension, or asthma and was not currently under any medication. His vitals were stable.

**Figure 2:**
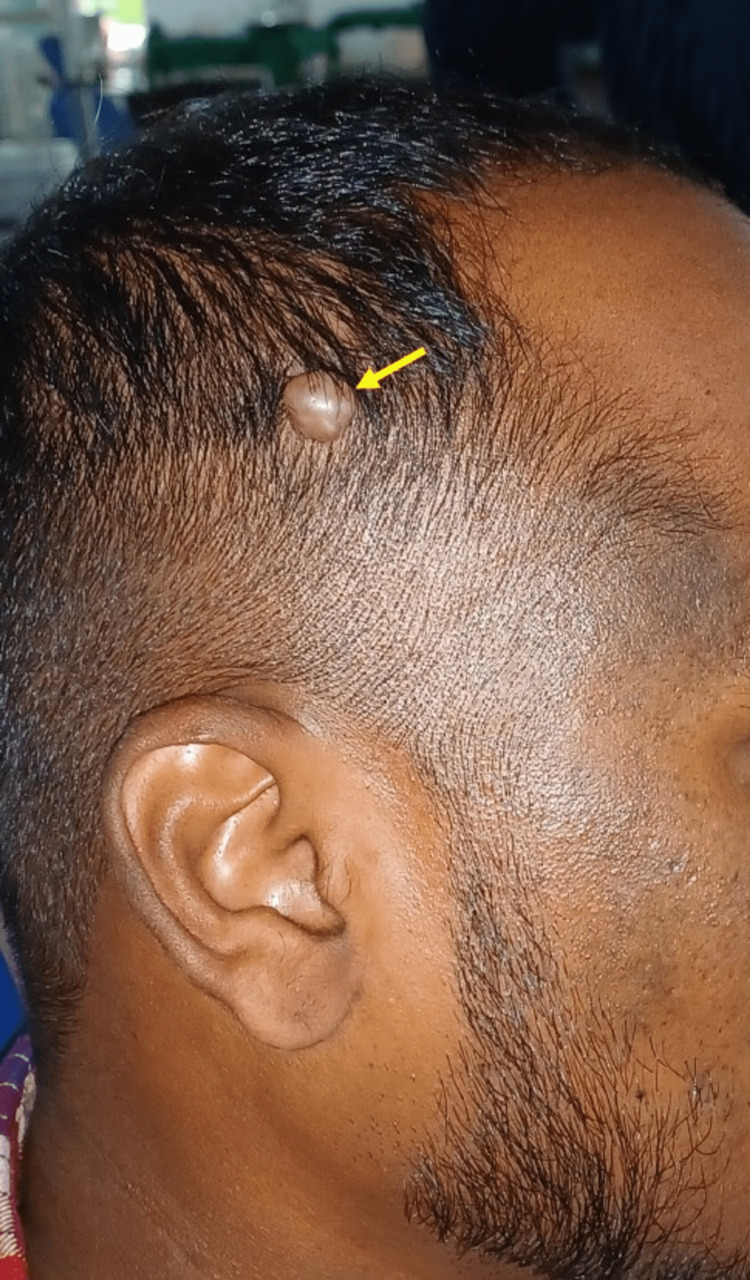
Extraoral examination also revealed a swelling on the right side of scalp

Further extraoral examination revealed swelling on the right side of his scalp. History revealed the swelling started as a small one and has progressed to the present size, not associated with malaise or fever. No history of similar swellings on any other parts of his body. Inspection revealed the bump was ovoid-shaped, approximately 1.5 x 1 cm in diameter, well-defined outline, and extended superiorly 4.5 cm above the helix of the right auricle and 6cm away from the right forehead region. The skin over the surface of the swelling was smooth, stretched, and appeared slightly paler than the adjacent area, non-pinchable, and shiny with no other secondary changes like punctum or pin-point sinus opening (Figure 2).

On palpation, the swelling is soft in consistency, non-tender, sessile, non-pedunculated, fluctuant, and compressible but not reducible (Video 1).

**Video 1:** Clinical examination of swelling on the scalp

**Figure 3:**
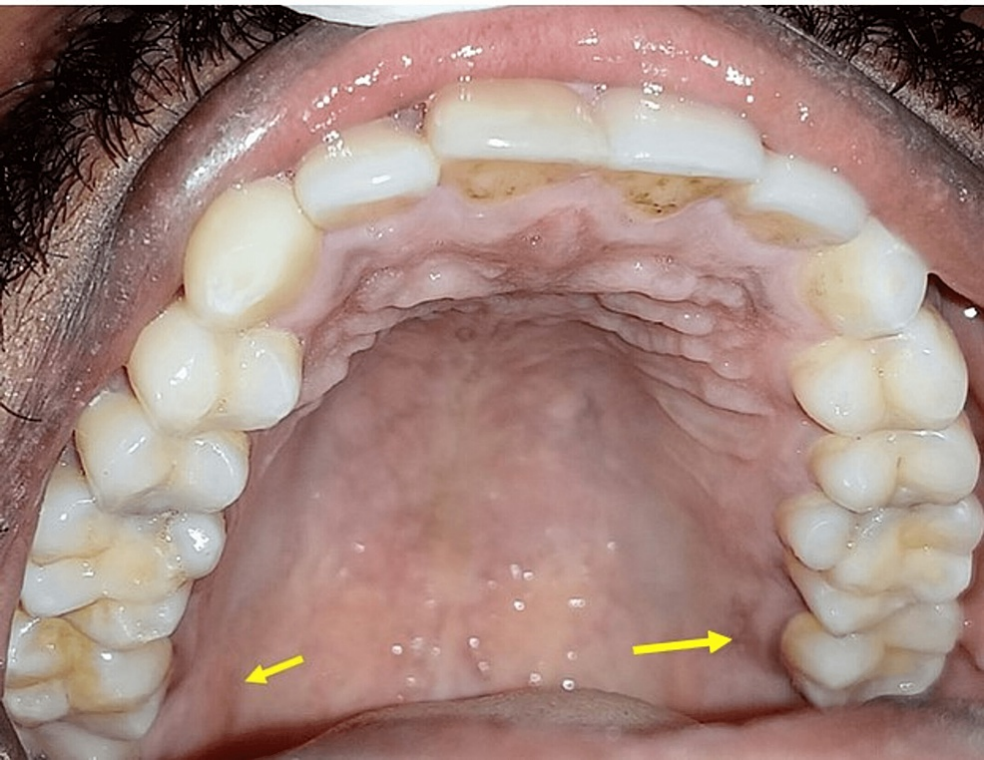
Intraoral examination revealed a discrete greyish pigmentation on both the right and left posterolateral region of the hard palate

Intraoral examination revealed discrete pigmentation on the right side of the posterolateral portion of the hard palate (Figure 3).

A provisional diagnosis of trichilemmal cyst and nevus of Ota was made based on the above clinical findings. The differential diagnosis considered were sebaceous cyst and dermoid cyst. Sebaceous cysts are characterized by the presence of punctum on their surface whereas trichilemmal cyst do not have a characteristic punctum. Dermoid cysts occur more near the midline along the line of fusion of various processes during the development of the head and neck. Trichilemmal cysts occur away from the midline usually on the lateral aspect of the scalp.

Case two

**Figure 4:**
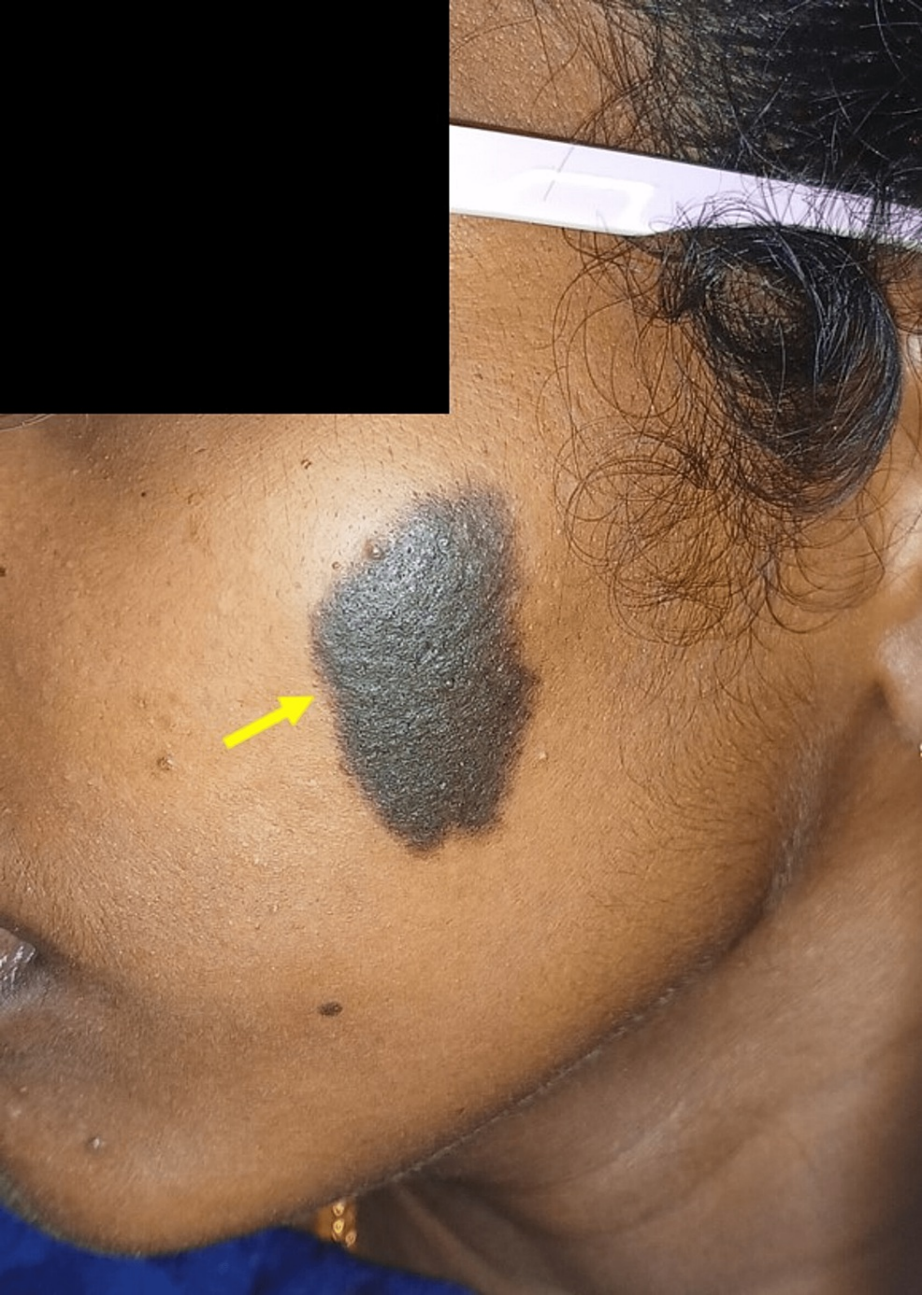
Extraoral examination revealed a well-demarcated dark greyish pigmented area on the left cheek region

A 27-year-old female reported to our dental outpatient department for a routine dental checkup. A general examination revealed her vitals were stable. Extraoral examination revealed an unusually large area of flat dark grey-brown intensely pigmented area on her left cheek region, measuring approximately about 4.5 x 3 cm in diameter (Figure 4).

History revealed the pigmented area was present from birth and asymptomatic. Her family history was non-contributory. No other similar pigmentations on any part of her body. On palpation, it is non-tender, and no other abnormal discharge is seen. Her biological values were within normal ranges. The ophthalmic evaluation revealed she is currently wearing spectacles to correct her shortsightedness (myopia) and did not reveal any other abnormalities. Our patients refused treatment for the pigmentations as they were completely asymptomatic in both cases.

Case three

**Figure 5:**
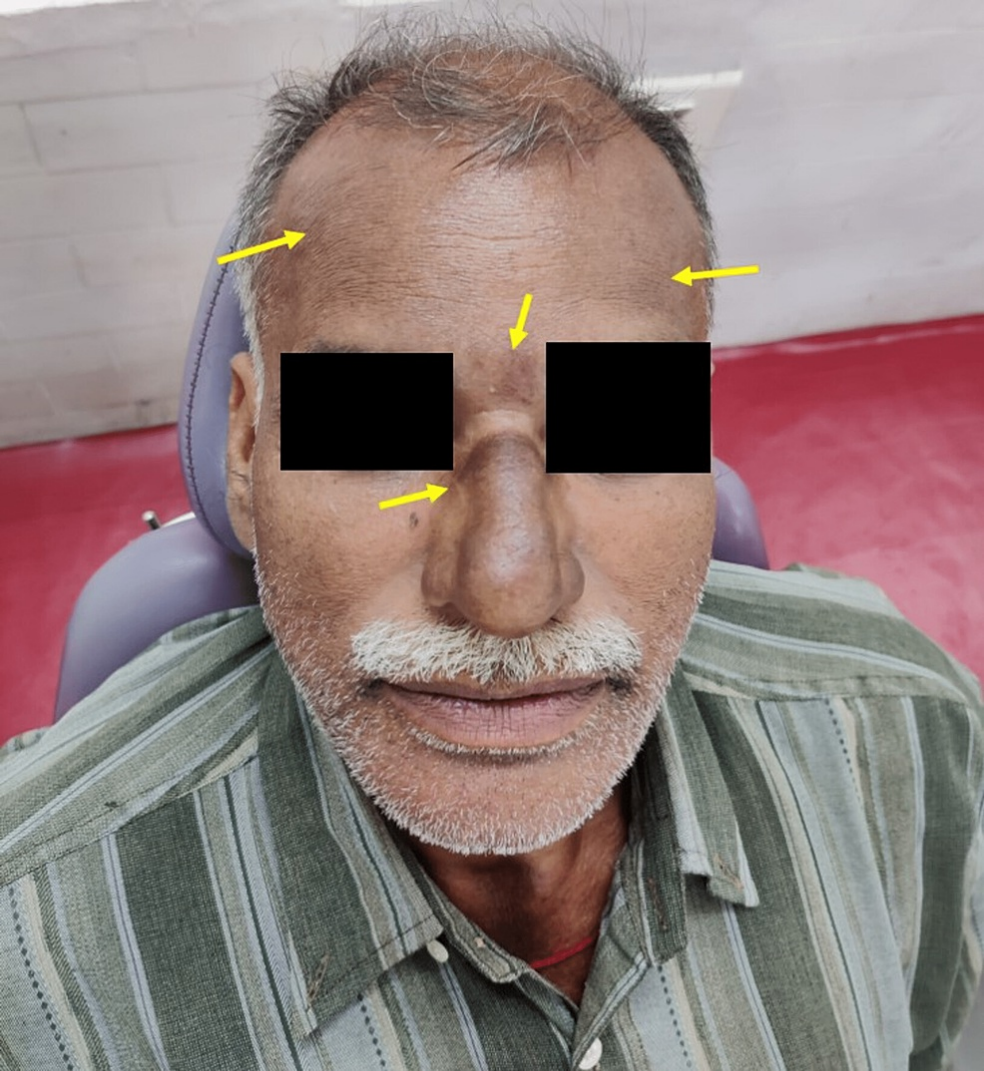
Extraoral examination revealed macular greyish pigmentations involving the nose, right and left forehead regions

A 47-year-old male presented to our department for a routine dental checkup. Extraoral examination revealed a discrete pigmentation involving the nose and right and left forehead regions (Figure 5).

**Figure 6:**
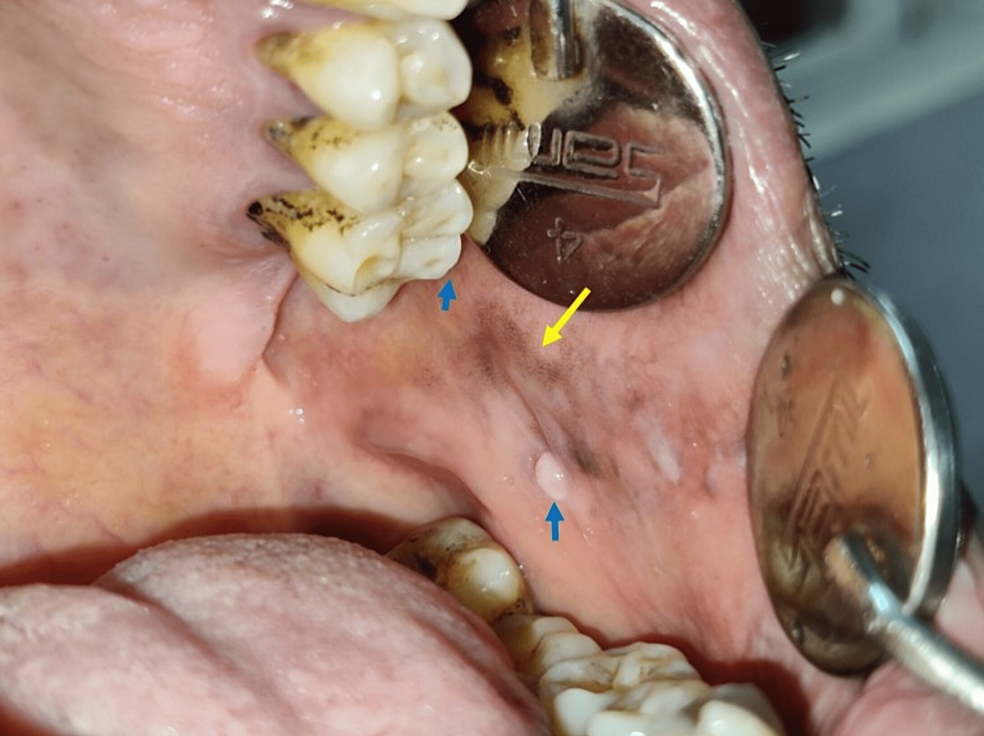
Intraoral examination revealed discrete flat greyish pigmentations on left buccal mucosa and a fibroma caused by sharp attrited distobuccal cusp of 26

History revealed the pigmentations on his nose had been present since his childhood days and completely asymptomatic. His family history was non-contributory. Further intraoral examination revealed discrete greyish pigmentation on his left buccal mucosa and a fibroma caused by irritation secondary to attrited distobuccal cusp 26 (Figure 6). The patient refused treatment for the pigmentations on his nose.

Correlating to the above clinical findings, a provisional diagnosis of nevus of Ota and irritational fibroma on the left buccal mucosa caused by sharp attrited distobuccal cusp 26 was made. There was no loss of visual acuity in this case; hence no special investigations for ophthalmic evaluation, such as optical coherence tomography, were performed. Coronoplasty of sharp attrited distobuccal cusp 26 was performed to relieve the chronic source of irritation.

## Discussion

Etiopathogenesis

Cysts of trichilemmal origin arise from abnormalities in the external root sheath of the hair follicle following a genetic mutation [2]. It occurs predominantly on the scalp, with a high concentration of hair follicles [2]. The pathological changes observed in the cyst may result from a) a rupture of the cyst and the resultant inflammation; or b) a balance between matrix metalloproteinases and their inhibitors (i.e., tissue inhibitors of metalloproteinases, or TIMPs), which are secreted primarily by macrophages that are activated [2]. These alterations, however, have not been fully explained by physical or immunological mechanisms [2]. In addition, adipocytokines, which are bioactive molecules, are secreted by the adipose tissue [2]. The adipose tissue produces anti-inflammatory molecules, such as adiponectin; adiponectin may regulate inflammation in cyst areas, where matrix metalloproteinases (MMPs) and TIMPs are abundant [2]. A final hypothesis is that pilar cyst pathophysiology is also mediated by signaling pathways involving zeta-chain-associated protein kinase (ZAP)-70 and p27, and that these antibodies are expressed in the etiopathogenesis of pilar cysts [2].

Clinical features

The incidence of pilar cysts is about 10% in the general population [2]. Most cases are sporadic and non-syndromic [2]. These are the most common types of skin cysts [2]. There is a higher incidence of these cysts in areas with a high concentration of hair follicles [2]. It is the most common cutaneous cyst on the scalp and the second most common on the head and neck [2]. In contrast to malignant lesions, pilar cysts are benign [2]. Approximately 2% of pilar cysts progress into malignant tumors, referred to as proliferating trichilemmal cysts or tumors [2]. It is possible to inherit trichilemmal cysts through autosomal dominant inheritance [2]. Typically, patients with familial pilar cysts are younger and have multiple lesions simultaneously [2]. These cysts develop from the epithelium between the sebaceous gland and the arrector pili muscle [2]. Most commonly, they occur on the head, especially the scalp [2]. It takes several years for the pilar cyst to grow to its full size; it grows very slowly [2]. Nevus of Ota was originally described as nevus fusco-caeruleus ophthalmo-maxillaris by Ota and Tanino in 1939 [3]. Nevus of Ota is caused by an increase in the number of melanocytes (melanocytosis) near the reticular layer of the dermis and is most commonly found in Japanese [3]. It is a benign dermatosis nevus of Ota was first described by Hulke in 1836 in a patient with unilateral cutaneous hyperpigmentation with malignant melanoma of the sclera and later in a case series in 1939 by Ota and Tanino in 26 Japanese patients [3]. About 60% are found at birth, 40% at puberty [3]. It is a congenital dermal hyperpigmentation syndrome that usually occurs in the first and the second division of the trigeminal nerve involving the sclera of eyelids, conjunctiva, choroid, optic nerve, and adjacent skin [3]. Nevus of Ota is usually benign but has a low malignant potential [3]. Clinically, it is associated with different types of vascular diseases such as large vessel arteritis like Takayasu’s arteritis, vascular defects like Klippel-Trénaunay syndrome, and Sturge Weber's syndrome [3]. The etiopathogenesis of nevus of Ota needs to be clarified [3]. Some hypothesized that nevus of Ota is formed by an alteration of the dorsolateral migratory pathway, and melanocytes from the neural crest during the second to the eighth gestation week of embryogenesis may be responsible for its formation-the entrapped melanocytes within the skin's dermis cause the characteristic gray-blue hyperpigmentation [3]. Next-generation sequencing performed on DNA in nevus of Ota from enucleated specimens revealed GNAQ /GNA11 mutations a rare GNAQ R183Q gene and a PMS1 truncation mutation [3]. Unilateral presentations of nevus of Ota are more common [3]. As a result of the entrapment of melanocytes, the conjunctiva and sclera, along with ipsilateral facial skin, become grey-blue [3]. These cases are at higher risk of developing uveal melanoma and glaucoma [3]. Occasionally, the palatal region is also affected [3]. Congenital oculodermal melanosis (ODM) is a congenital, non-hereditary nevus, although the degree of pigmentation may increase with adolescence, adulthood, aging, and pregnancy [3]. The lesion appears more bluish or brownish due to the Tyndall phenomenon or effect caused by the reflectance of light by melanocytes in the reticular dermis [4]. The lesion may present a wide range of coloration due to the accumulation of melanocyte cells in a stratified manner in the dermis [4]. 

Classification of nevus of Ota

Mishimas classification of nevus of Ota is enumerated [4] (Table 1).

**Table 1:** Mishimas Classification of nevus of Ota [4]

Subtypes	Intensity	Pigmentation	Area involved
Type I	(mild)	Light brown / Grey	Periocular,zygomatic region,forehead, nose
Type A	(mild)	Light brown / Grey	Type A is periocular.
Type B	(mild)	Light brown / Grey	Type B involves the zygomatic region
Type C	(mild)	Light brown / Grey	Type C involves the forehead.
Type D	(mild)	Light brown / Grey	Type D involves the only nose.
Type II	(Moderate)	Deep slate Grey	Similar to type I but worse in severity
Type III	(Intensive)	Deep blue to brown	Periocular, nose, and scalp involvement.
Type IV	(Intensive)	Dark brown	Bilateral involvement.

Epidemiology

Nevus of Ota or oculodermal melanosis (ODM) affects 0.014% to 0.034% of the Asian population, most commonly present at birth [5]. The incidence in Asians is 1-2 per 1000 [5]. 

The classification of nevus of Ota by Taninos is discussed [5] in (Table 2).

**Table 2:** Tanino classification of nevus of Ota [5]

Type	Distribution of Nevi
Type IA. Mild orbital type	Distribution over the upper and lower eyelids, periocular and temple region
Type IB. Mild zygomatic type	Pigmentation is found in the infrapalpebral fold, nasolabial fold and the zygomatic region
Type IC. Mild forehead type	Involvement of the forehead alone.
Type ID	Involvement of ala nasi alone.
Type II Moderate type	Distribution over the upper and lower eyelids, periocular, zygomatic, cheek and temple regions.
Type III	The lesion involves the scalp, forehead, eyebrow and nose
Type IV. Bilateral type	Both sides are involved.

A proposed clinical modification of Tanino's classification of nevus of Ota by Mukhopadhyay AK is discussed [5] in (Table 3).

**Table 3:** Modified Tanino's Classification of nevus of Ota [5]

Type	Distribution of Nevi
Type IA. Mild orbital type	Distribution over the upper and lower eyelids, periocular and temple region
Type IB. Mild zygomatic type	Pigmentation is found in the infrapalpebral fold, nasolabial fold and the zygomatic region
Type IC. Mild forehead type	Involvement of the forehead alone.
Type ID	Involvement of ala nasi alone.
Type II Moderate type	Distribution over the upper and lower eyelids, periocular, zygomatic, cheek and temple regions.
Type III	The lesion involves the scalp, forehead, eyebrow and nose
Type IV. Bilateral type	Both sides are involved.
Type VA	Unilateral nevus of Ito without nevus of Ota.
Type V B	Bilateral nevus of Ito without nevus of Ota.
Type VI A	Unilateral nevus of Ota with unilateral nevus of Ito (ipsilateral and contralateral)
Type VI B	Bilateral nevus of Ota with unilateral nevus of Ito (ipsilateral and contralateral)
Type VI C	Unilateral nevus of Ota (ipsilateral and contralateral) with bilateral nevus of Ito
Type VI C- E	A letter “E” may be suffixed with the class (e.g., type VIC-E etc.) the nevi may be associated with extra cutaneous manifestations wherever it is applicable.

Differential diagnosis

Sun's nevus is often bilateral, not unilateral like the nevus of Ota, and they differ from the nevus of Ota in that they have pigmentation distribution along the territory distribution of lateral cutaneous brachial nerves and posterior supraclavicular nerves [5]. An oral melanotic macule typically occurs on the palate but is smaller [6]. Nevus of Ito usually involves the posterior supraclavicular nerve and lateral cutaneous nerve of the shoulder [6]. A nevus of Horis or acquired bilateral nevus of Ota-like macules differs from a nevus of Ota by its bilateral occurrence, later onset, and lack of scleral involvement [6]. The extracutaneous involvement of nevus of Ota includes nasal mucosa (30%), tympanum (55%), palate (20%) and pharynx (25%) [6]. The differential diagnosis of Trichilemmal cyst includes dermoid cyst, epidermoid inclusion cyst, Pilomatrixoma, cutaneous lipomas, steatocytoma multiplex, and Favre Racochout syndrome [6]. A proliferating trichilemmal tumor is a solid cystic neoplasm that shows trichilemmal differentiation similar to the isthmus of hair follicles [6]. This tumor has been called by various names in literature like sub epidermal acanthoma, epidermoid carcinoma in the sebaceous cyst, giant hair matrix tumor proliferating epidermoid cyst, invasive hair matrix tumor of the scalp, trichochlamydocarcinoma, proliferating trichilemmal cyst, proliferating pilar cyst, proliferating follicular cystic neoplasm, proliferating isthmica cystic carcinoma proliferating trichilemmal cystic squamous cell carcinoma [6]. The complications of trichilemmal cysts include cosmetic disfigurement, infection, calcification, and rarely malignant transformation (2%) [6]. The post-surgical complications of trichilemmal cysts include infection, bleeding, and scarring [6].

The pilomatricoma is most commonly found in the extremities, particularly the neck and head, whereas the Trichilemmal cyst is more commonly found on the scalp [7]. Ultrasonography revealed complete patterns and scattered dots in pilomatricomas, but lump-like patterns in trichilemmal cysts [7]. A trichilemmal cyst is more likely to appear as a mass with a hypoechoic rim and peritumoral hyperechogenicity than a pilomatricoma [7]. In trichilemmal cysts, posterior enhancement was significantly more common, whereas in pilomatricomas, posterior shadowing was more common [7]. The posterior shadowing associated with pilomatricomas may be more common because pilomatricomas have internal calcification, ossification, or both, especially in a complete pattern that is highly reflective or attenuating [7]. There is no vascularization detected on a color Doppler Ultrasonography (US) of any trichilemmal cyst, while most pilomatricomas are vascular [7]. A pilomatricoma should be considered if one of these four US features is absent: a) an absence of complete internal echogenic foci, b) an absence of a hypoechoic rim, c) an absence of peritumoral hyperechogenicity, and d) an absence of vascularity [7].

A rare genetic disorder, steatocystoma multiplex has an autosomal dominant inheritance pattern and is characterized by hamartomatous malformation of the pilosebaceous duct junction caused by a mutation in keratin k7 gene and characterized by multiple asymptomatic cysts on the axilla, groin, trunk, sternum of males, the scrotum, and the proximal extremities [8]. The face and scalp are rarely affected [8]. Early steatocystoma multiplex lesions are dome-shaped and translucent, but they become yellowish with time. Inflammation and scarring are the results of spontaneous rupture [8]. There is keratin contained in trichilemmal cysts and sebaceous lobules between the cyst wall and the pilar canal in steatocystomas [8]. A trichilemmal cyst is formed by the outer root sheath of the follicle, an epidermal cyst by the infundibulum, and a vellus hair shaft by the infundibuloisthmic junction [8]. Combined treatments of Er:YAG laser, radiofrequency incision probes, and cryotherapy are effective in treating steatocystoma multiplex [8].

The Favre Racochout syndrome is characterized by yellowish hues and large, open, black comedones scattered across the temporal and periorbital areas, rarely on retro auricular, forearm, earlobes, lateral necks, deep wrinkles, and furrows over a background of atrophic, actinically damaged skin. In the nose and periocular region, multiple small papules, nodules, and cystic lesions develop bilaterally symmetrically [9].

The various literature reviews on various cases of nevus of Ota reported (Table 4).

**Table 4:** The various literature reviews of case reports on nevus of Ota

Author	Year	Age / Gender	Location	Clinical Description
Yamada-Kanazawa et al. [10]	2022	2 month / Girl	Left auricle	Nevus of Ota on the auricle successfully treated with Q-switched ruby laser
Mularoni et al. [11]	2021	27-year-old Male	Right Periocular region	Superficial sclerotomy showed cosmetic improvement in patients with nevus of Ota with periocular pigmentation
Taneja et al. [12]	2021	28-year-old Female	The left side of the face	Nevus of Ota along with Plexiform Neurofibroma
Maguire et al. [13]	2019	48-year-old Female	The left side of the face, Left eye, and Left buccal mucosa	Nevus of Ota or "oculodermal melanocytosis" is a rare congenital hamartoma of dermal melanocytes causing blue-gray hyperpigmentation of the eye and surrounding structures. The condition mainly affects the ophthalmic and maxillary divisions of the trigeminal nerve.
Toomey et al. [15]	2019	34-year-old Female	Bilateral nevus of ota and uveal melanomas	Nevus of ota is associated with uveal melanomas
Rishi et al. [16]	2015		Nevi of ota in all three divisions of trigeminal nerve on the left side of the face and left eye	Nevus of Ota along with conjunctival melanocytosis, anterior and intercalary staphyloma, leucomatous corneal opacity, and pseudo proptosis
Mukhopadhyay [5]	2013	24-year-old male	The right side of his face and palate	Nevus of ota is a rare dermal melanosis
Sharma et al. [17]	2011	22 / Male	The left side of the face and palate	Palatal pigmentation usually blends with oral mucosa and is typically irregular, ill-defined, and often mottled patch
Baroody et al. [18]	2004	65-year-old Male	Left Preauricular region	An alteration in the concentration of glucosaminoglycans favors the differentiation of neural crest cells into the melanocytic phenotype. The tissues are characterized by increases in the number and size of these melanocytes.

Investigations

Investigation modalities if nevus of Ota occurs along with pigmentations in the sclera and skin [19] (Table 5). 

**Table 5:** Investigation modalities for ophthalmic and dermatological evaluation for nevus of Ota with episcleral pigmentations [19]

Evaluation	Diagnostic tests
Eye	Ultrasonography (USG), Magnetic Resonance Imaging (MRI), Positron Emission Tomography (PET)
Anterior segment	Anterior segment optical coherence Tomography (ASOCT)
Posterior segment	Fundus autofluorescence
	Fluorescein and Indocyanine green angiography
	Spectral Domain Optical Coherence Tomography (SDOCT)
Fundus	Fundoscopy
Sclera	Slit-lamp examination
Retina	Optical Coherence Tomography
Skin	Dermatoscope
	Epiluminescence Microscopy

Treatment modalities

Q-switched Nd

YAG and Ruby lasers have shown promising results in treating Ota nevus [20]. These lasers' mechanism of action is based on selective photothermolysis, which combines a photothermal effect with a photomechanical or photoacoustic reaction [20]. Trichilemmal cysts are best treated by surgical excision under local or general anesthesia.

## Conclusions

Dentists must also have a sound knowledge about nevus of Ota and trichilemmal cysts. A cystic swelling on the scalp must not always be overlooked as a sebaceous cyst, as trichilemmal cysts often clinically mimic sebaceous cysts and do not have a punctum on the surface. Dentists must have a sound knowledge of nevus of Ota and trichilemmal cysts, which can cause psychological impact and affect a patient's quality of life. A multidisciplinary team approach involving a dentist, dermatologist, and retinal specialists is needed to manage patients with nevus of Ota with trichilemmal cyst.
